# Mental and social wellbeing trajectory during the pandemic for vulnerable populations

**DOI:** 10.3389/fpubh.2024.1337401

**Published:** 2024-04-08

**Authors:** Andrew Joyce, Thach Tran, Ruby Stocker, Jane Fisher

**Affiliations:** ^1^Centre for Social Impact Swinburne, Swinburne University of Technology, Melbourne, VIC, Australia; ^2^Research & Impact, VicHealth, Melbourne, VIC, Australia; ^3^Global and Women’s Health, Public Health and Preventive Medicine, Monash University, Melbourne, VIC, Australia

**Keywords:** coronavirus restrictions, vulnerable populations, wellbeing trajectory, mental health, social connectedness

## Abstract

**Objectives:**

We investigated changes over time in mental and social wellbeing indicators for vulnerable population subgroups during the pandemic. These groups were younger people, people with disabilities, low-income groups, unemployed, culturally, and linguistically diverse communities (CaLD), and Aboriginal and Torres Strait Islander peoples.

**Methods:**

A series of four repeated population representative surveys were conducted in June 2020, September 2020, January 2022, and June 2022. Questions included items on psychological distress, financial hardship, social connection, and life satisfaction.

**Results:**

For most groups, social connection and life satisfaction improved in 2022 relative to 2020. Psychological distress and financial hardship showed the opposite pattern, with some groups having worse results in 2022 relative to 2020. People without any vulnerability had better mental health and social wellbeing outcomes at each time point relative to the vulnerable population subgroups.

**Conclusion:**

Pandemic-related policies had differential effects over time and for different population groups. Future policies and research need to closely monitor how they impact population subgroups, and the overall results clearly demonstrate the inequity in mental health and social wellbeing outcomes for vulnerable population cohorts.

## 1 Introduction

In the COVID-19 pandemic, it was recognized very early that the disruption and stress caused by the virus and the fear of infection would have considerable mental health and social wellbeing impacts. In Australia, a number of studies have examined the psychological distress levels of the adult population and certain communities (1–10). Most of these studies were cross-sectional and used a specific measure of depression or anxiety, i.e., the Kessler Psychological Distress Scale. There was a considerable range in data on the prevalence of high psychological distress, from 17.7 to 48.3% (2, 6), with approximately one-third experiencing psychological distress as a common finding. Some of the risk factors for higher levels of psychological distress included being of a younger age, being female, having a pre-existing mental health condition, and having a fear of COVID-19 (1, 2, 4, 6, 9, 10). Some of the protective factors were exercise and older age (3, 6, 9).

Subjective wellbeing was another way in which wellbeing across the population during COVID-19 was assessed. The methods of these studies were again mainly cross-sectional online surveys using a variety of wellbeing indexes and scales, including the Personal Wellbeing Index (6, 11–18). Characteristics potentially related to subjective wellbeing investigated included gender, age, education level, employment, impact of COVID-19 on the study, urban vs. rural residence, mental and physical health comorbidities, financial stability, and physical activity (6, 11–18). Some of the risk factors for reduced scores on the wellbeing scales included being female, of a younger age, and having a mental health condition (12, 14–16, 18). Factors related to higher scores on the wellbeing scales included older age and financial stability (12, 16, 18).

Most of this research has been based on cross-sectional surveys either at one time period or comparing results to previous surveys conducted prior to COVID-19, and many of these studies relied on convenience samples. While the existing research has revealed significant differences between population groups at single time points, there is a lack of research understanding how the mental health and wellbeing of these groups changed over time during the pandemic. Comparing survey results before and during the pandemic revealed that changes in mental health status were not uniform. One study showed that the decline in mental health was worse for men, young Australians, and those who were employed (2).

To understand how the pandemic impacted various groups in Victoria, a series of four repeated surveys were conducted by the Victorian Health Promotion Foundation (VicHealth), a health promotion state-based government organization in Victoria, Australia. These surveys aimed to inform VicHealth and its stakeholders on how to support the health and wellbeing of Victorians both during and after the pandemic. The four surveys were undertaken in 2020 and 2022 using similar methods. Findings published in 2022 from the first two surveys found that there was a drop in life satisfaction but no change in psychological distress overall; however, younger age was associated with an increased risk of psychological distress (19).

Previous research suggests that certain groups may be at an increased risk of experiencing mental health and wellbeing impacts from the pandemic and should be monitored more closely. This includes younger people, people with disabilities, low-income groups, the unemployed, culturally and linguistically diverse communities (CaLD), and Aboriginal and Torres Strait Islander peoples. This study aimed to examine the mental health and wellbeing trajectory of these population subgroups during the COVID-19 pandemic using data from the four VicHealth surveys.

## 2 Method

### 2.1 Study setting

#### 2.1.1 About Victoria

Victoria, with a population of approximately 6.6 million people in 2022, is the second most populous state among the eight states and territories of Australia (20). The first case of COVID-19 in Australia reported on 25 January 2020 was in Victoria. Most states and territories implemented lockdowns in 2020, but Victoria also had a number of lockdowns in 2021. The Victorian Government implemented the first lockdown on 30 March 2020 and several other lockdowns throughout 2020 and 2021 according to these approximate timelines:

- 30 March to 12 May 2020 (43 days)
- 8 July to 27 October 2020 (111 days)
- 12 to 17 February 2021 (5 days)
- 27 May to 10 June 2021 (14 days)
- 15 to 27 July 2021 (12 days)
- 5 August to 21 October 2021 (77 days)

These restrictions included border closures, stay-at-home orders, school closures, restrictions on social gatherings, mask-wearing, and curfews, among other strategies.

The federal and state governments introduced several new financial support programs for businesses and individuals affected by the pandemic, including coronavirus restrictions. The most important program for employers and employees was the JobKeeper payment, which helped to support businesses and their employees affected by the economic impact of the pandemic (21). In the first phase of JobKeeper payment, implemented from 30 March to 27 September 2020, eligible businesses and not-for-profits were able to receive $1,500 per fortnight per employee to cover the cost of wages. The extension phase of JobKeeper payment was from 28 September 2020 to 28 March 2021 targeting businesses significantly affected by the economic downturn.

Households and individuals were supported by social assistance benefits in cash, including the Coronavirus Supplement and Economic Support Payment. The Coronavirus Supplement, which started on 27 April 2020 and ended on 31 March 2021 provided $275 a week for people in some vulnerable groups, including people who were receiving sickness allowance, parenting payments, or farm household allowance, and young adults who were receiving JobSeeker Payment and Youth Allowance. The Economic Support Payment was a one-off payment of $750 from March 2020 to July 2020 and $250 from December 2020 to March 2021 for families with low income or having a concession card.

### 2.2 The VicHealth coronavirus Victorian wellbeing impact surveys

A series of four repeated surveys were conducted by VicHealth. All Victorian residents aged 18 years and older were eligible for surveys 1 (conducted in June 2020) and 2 (September 2020). In surveys 3 (January 2022) and 4 (June 2022), the age range was expanded to 16 years and above. Participants were recruited using LiveTribe, a research-only panel operated and managed by i-Link Research (22). Panelists of the LiveTribe are recruited through a range of strategies, including print media, online marketing initiatives, direct mail, social media platforms, affiliate partnerships, and personal invitations. Respondents to these surveys received a nominal incentive for their participation, according to the panel policies. The surveys were completely anonymous and built using a web-based platform for online self-completion. All participants provided online written informed consent.

### 2.3 Participants

In the first two surveys, sample sizes were 2000. The sample sizes were increased to 2,500 for each of the last two surveys to include participants aged 16 and 17. However, in this study, we included only data from respondents aged 18 years and older from all four surveys to be consistent across the surveys.

In each survey, respondents were asked if they would be happy to be contacted for this survey again in the future. The ones agreeing to be recontacted were invited to participate in the subsequent surveys. Finally, 1,008 participants provided data at both surveys 1 and 2; 432 participants provided data at both surveys 2 and 3; and 731 participants provided data at both surveys 3 and 4. A process of weighting using key demographic variables was undertaken to ensure the sample represented population norms. Further information about the weighting procedure can be found in the VicHealth report (23).

### 2.4 Data sources

#### 2.4.1 Life satisfaction

For the measure of life satisfaction, participants rated from 0 (completely dissatisfied) to 10 (completely satisfied) how satisfied they were with their life as a whole. Low to medium life satisfaction was defined as a score of 6 or lower, consistent with the scoring from the Victorian Population Health Survey.

#### 2.4.2 Psychological distress

Psychological distress was assessed using the Kessler Psychological Distress Scale – 6 Item version (K6) (24). K6 is widely used in research worldwide, including in Australia, to assess the symptoms of serious mental illness in the general population. The K6 comprises 6 questions about a person’s emotional state in the last month, namely feeling nervous; hopeless; restless/fidgety; so depressed that nothing could cheer up; that everything was an effort; and worthless. Each question has five options scored (Australian scoring method) from 1 (none of the time) to 5 (all of the time). The scale scores are the sum of the six question scores and range from 6 to 30. The higher scores indicate higher levels of psychological distress.

K6 has been evidenced to perform well against the World Health Organization’s Composite International Diagnostic Interview (CIDI) for depressive and anxiety disorders among Australian adults (the areas under receiver operating characteristic curves of 0.89, 95%CI: 0.88 to 0.90) (25). A threshold of 19 or higher K6 scores in the Australian scoring method indicates high psychological distress or probable serious mental illness (26).

#### 2.4.3 Social connection

There was one item where respondents were asked to rate the degree to which they agreed with the statement ‘I feel connected with others’. The options are (1) strongly disagree; (2) disagree; (3) mildly disagree; (4) mildly agree; (5) agree; and (6) strongly agree.

#### 2.4.4 Financial hardship

Financial hardship was assessed using a 9-item scale, asking whether they experienced certain events because of a shortage of money. This scale comprises six items developed by the Australian Bureau of Statistics for use in national population-based income and expenditure surveys prior to the COVID-19 pandemic and three study-specific items (25). The six items developed by the Australian Bureau of Statistics are:

1. Could not pay electricity, gas, or telephone bills on time
2. Could not pay the rent or mortgage on time
3. Pawned or sold something
4. Went without meals
5. Asked for financial help from friends or family
6. Asked for help from welfare/community organizations

The three study-specific items are:

7 Attended a food relief agency, food bank, or food pantry (or similar) to access food relief
8 Skipped a meal in order to feed your household
9 Ran out of food and could not afford to buy more

In surveys 1 and 2, participants were asked about their experiences from the start of the COVID restrictions until the survey date. In surveys 3 and 4, participants were asked about their experiences from October 2021 (when the final COVID restrictions were eased) until the survey time.

#### 2.4.5 Socio-demographic characteristics

Socio-demographic characteristics were collected using study-specific questions: age, gender, residential postcode, being an Aboriginal and Torres Strait Islander, country of birth, speaking a language other than English at home, highest education level, having any disability, household composition, main activity, and income. The residential postcode was used to identify the residential region (Melbourne, the capital city of Victoria, or other) and the Index of Relative Socio-economic Advantage and Disadvantage (SEIFA) using the most recent Australian Bureau of Statistics data (27).

#### 2.4.6 Data analyses

We created six vulnerable groups:

1. Younger people: age < 25 years.
2. People with disabilities: reported any disability.
3. Low income: < $40,000 per year.
4. Unemployed: people who currently are not in a paid job but are looking for work.
5. CaLD communities: speaking a language other than English at home and/or born in a non-English speaking country.
6. Aboriginal and Torres Strait Islander peoples: who endorsed Aboriginal or Torres Strait Islander origin

We also divided the samples into two groups: people with any of the vulnerabilities mentioned above and people without any of the vulnerabilities mentioned above.

The outcomes (i.e., low to medium life satisfaction; K6 score; high psychological distress; agreeing with the statement ‘I feel connected with others’; and having any financial difficulty) were estimated (proportions and 95%, or mean and 95%, where appropriate) for each of the subgroups described above.

#### 2.4.7 Ethical consideration

Ethics approval for Surveys One and Two was provided by the Australian National University Human Research Ethics Committee (2020/264) on 20 May 2020. Ethics approval for Surveys Three and Four was provided by the Bellberry Human Research Ethics Committee (2021-11-1312) on 6 January 2022.

## 3 Results

The numbers and proportions of participants among the vulnerable groups across the samples are presented in Table 1.

**Table 1:** Numbers and proportions of participants by the vulnerable groups across the survey samples.

	Survey 1	Survey 2	Survey 3	Survey 4
Vulnerable group				
Aboriginal and Torres Strait Islander peoples	61 (3.1)	61 (3.1)	88 (3.7)	111 (4.6)
People with disabilities	405 (21.3)	408 (21.6)	539 (22.7)	530 (22.1)
Young (<25 yrs)	256 (12.8)	247 (12.4)	638 (27.3)	616 (25.4)
CaLD	493 (24.7)	443 (22.2)	617 (24.8)	554 (22.2)
Low income (< $40,000 per year)	497 (29)	485 (27.9)	622 (28.9)	591 (26.3)
Unemployed	138 (6.9)	152 (7.6)	196 (7.86)	145 (5.8)
People with any vulnerability above	1,220 (61)	1,192 (59.6)	1,684 (67.55)	1,638 (65.52)
People without any vulnerability above	780 (39)	808 (40.4)	809 (32.45)	862 (34.48)

The results for low to medium life satisfaction (Table 2) broadly show a similar pattern between people with and without vulnerability; however, the starting positions are different. Life satisfaction increased more for people without any vulnerability between 2020 and 2022. There was a 10% higher rate of dissatisfaction in the group with a vulnerability, and this difference was maintained for each survey. The trend was similar for the two groups, with a peak of dissatisfaction at survey 2, which had improved by survey 4. What is interesting to note about the results is that survey 2 was the only data collection that occurred during a lockdown. The trends for most subgroups were similar, apart from people with low incomes, where they were similar for each survey.

**Table 2:** Proportion (%) and 95% CI of people with low to medium life satisfaction.

	Survey 1	Survey 2	Survey 3	Survey 4
Vulnerable group				
Aboriginal and Torres Strait Islander peoples	60 (46.5; 72.4)	57.4 (44.1; 70)	46 (35.2; 57)	37.6 (28.5; 47.4)
People with disabilities	61.4 (56.4; 66.2)	66.3 (61.4; 70.9)	62.1 (57.9; 66.3)	53.3 (49; 57.7)
Young (<25 yrs)	53 (46.6; 59.2)	50.8 (44.4; 57.3)	48.8 (44.8; 52.8)	42.9 (39; 46.9)
CaLD	50.4 (45.9; 55)	52.7 (47.8; 57.4)	42.4 (38.4; 46.5)	37 (32.9; 41.2)
Low income (< $40,000 per year)	59.9 (55.4; 64.3)	61.6 (57; 65.9)	58.8 (54.7; 62.7)	55.7 (51.5; 59.7)
Unemployed	64.2 (55.4; 72.3)	69.9 (61.7; 77.2)	62.3 (55; 69.2)	53.6 (45; 62)
People with any vulnerability above	53.3 (50.4; 56.1)	56.5 (53.6; 59.4)	49.6 (47.2; 52)	42.3 (39.9; 44.8)
People without any vulnerability above	39.5 (36; 43)	50.9 (47.3; 54.4)	37.7 (34.4; 41.2)	32 (28.9; 35.3)

The results of psychological distress contrast interestingly with life satisfaction. Whereas life satisfaction improved in surveys 3 and 4, this was not the case for psychological distress (Table 3). For some groups, it was relatively stable or showed a non-significant increase. For other groups, the average score significantly increased in surveys 3 and 4 relative to surveys 1 and 2. The proportion of people experiencing psychological distress increased for Aboriginal and Torres Strait Islander peoples, people with disabilities, and people with low income from 2020 to 2022. Psychological distress also increased for Aboriginal and Torres Strait Islander peoples, but due to the smaller sample size, this increase was not significant. The results of the proportions that experienced high psychological distress (Table 4) show a significant gap between each subgroup and those without any vulnerability.

**Table 3:** Mean (95% CI) Kessler Psychological Distress Scale – 6 (K6) score.

	Survey 1	Survey 2	Survey 3	Survey 4
Vulnerable group				
Aboriginal and Torres Strait Islander peoples	16.3 (15; 17.6)	16.1 (14.8; 17.4)	18.7 (17.3; 20.2)	16.7 (15.8; 17.6)
People with disabilities	13.8 (13.1; 14.4)	14 (13.4; 14.6)	16.6 (16; 17.2)	15.1 (14.5; 15.6)
Young (<25 yrs)	14.4 (13.6; 15.1)	14.6 (13.8; 15.4)	15.3 (14.9; 15.8)	15.5 (15.1; 15.8)
CaLD	13.8 (13.3; 14.4)	13.6 (13.1; 14.2)	14.2 (13.8; 14.7)	13.6 (13.1; 14.1)
Low income (< $40,000 per year)	12.9 (12.4; 13.4)	13 (12.4; 13.5)	14.9 (14.4; 15.4)	14.3 (13.8; 14.8)
Unemployed	14.1 (13.1; 15.1)	15.3 (14.2; 16.3)	15.8 (14.9; 16.7)	15.2 (14.3; 16.2)
People with any vulnerability above	13.2 (12.9; 13.5)	13.3 (12.9; 13.6)	14.6 (14.3; 14.8)	14.1 (13.8; 14.3)
People without any vulnerability above	10.9 (10.6; 11.2)	11.4 (11; 11.7)	11.7 (11.3; 12.1)	11.6 (11.2; 12)

**Table 4:** Proportions (%) and 95% CI of high psychological distress (a cutoff score of 19 or more out of 30 is used here as an indicator of high psychological distress).

	Survey 1	Survey 2	Survey 3	Survey 4
Vulnerable group				
Aboriginal and Torres Strait Islander peoples	28.8 (17.8; 42.1)	26.2 (15.8; 39.1)	40.7 (29.9; 52.2)	32.4 (23.9; 42)
People with disabilities	25.3 (21.1; 29.9)	23.4 (19.3; 27.8)	37.8 (33.7; 42.1)	30.2 (26.3; 34.3)
Young (<25 yrs)	25.2 (19.9; 31.1)	26.7 (21.2; 32.8)	26 (22.6; 29.7)	25.4 (22; 29.1)
CaLD	22 (18.3; 26)	19.4 (15.8; 23.5)	23.1 (19.8; 26.8)	20.6 (17.3; 24.3)
Low income (< $40,000 per year)	18.1 (14.7; 21.8)	18 (14.6; 21.7)	28.2 (24.6; 31.9)	26 (22.4; 29.7)
Unemployed	23.9 (16.9; 32)	30.6 (23.3; 38.7)	32.5 (25.9; 39.6)	29.4 (22.1; 37.6)
People with any vulnerability above	19.4 (17.1; 21.7)	19.1 (16.8; 21.5)	24.8 (22.8; 27)	21.6 (19.6; 23.7)
People without any vulnerability above	7.7 (5.9; 9.8)	11.7 (9.5; 14.1)	13.6 (11.3; 16.2)	12.1 (10; 14.5)

The pattern of results for social connection was similar for each group, which showed an improvement from the two survey results of 2020 compared to 2022 (Table 5). The only exception was Aboriginal and Torres Strait Islander peoples, who had higher scores of social connection in 2020 relative to other groups and maintained these results in 2022. People with disabilities and people who were unemployed had much lower social connection scores for each survey.

**Table 5:** Social connection: proportions (%) and 95% CI of people agreed with the statement “I feel connected with others”.

	Survey 1	Survey 2	Survey 3	Survey 4
Vulnerable group				
Aboriginal and Torres Strait Islander peoples	72.4 (59.1; 83.3)	82 (70; 90.6)	76.7 (66.4; 85.2)	80.7 (72.1; 87.7)
People with disabilities	54.9 (49.8; 60)	53.4 (48.4; 58.4)	64.9 (60.6; 69)	65.4 (61.2; 69.5)
Young (<25 yrs)	67.4 (61; 73.3)	63.2 (56.7; 69.3)	79.5 (76.1; 82.6)	83.9 (80.7; 86.7)
CaLD	61.5 (56.9; 65.9)	58.4 (53.6; 63.1)	80.4 (76.9; 83.5)	80.3 (76.7; 83.6)
Low income (< $40,000 per year)	54.9 (50.3; 59.5)	52.3 (47.7; 56.9)	68.9 (65; 72.6)	69.2 (65.2; 72.9)
Unemployed	50.4 (41.4; 59.4)	50.3 (41.9; 58.8)	58.7 (51.2; 65.9)	60.7 (52.1; 68.9)
People with any vulnerability above	60.7 (57.8; 63.5)	57.3 (54.4; 60.2)	75 (72.8; 77.1)	76.8 (74.7; 78.9)
People without any vulnerability above	67.4 (63.9; 70.8)	57.3 (53.7; 60.9)	79.1 (76; 81.9)	82.1 (79.3; 84.6)

The results of financial hardship revealed that Aboriginal and Torres Strait Islander peoples experienced very high levels, as measured in each survey (Table 6). For every other group, there was a significant increase at survey 4 (and some at survey 3) compared to surveys 1 and 2. This also included people without any vulnerability, although they had significantly less financial hardship relative to the subgroups at each survey time point.

**Table 6:** Proportion (%) and 95% CI of people having any financial hardship.

	Survey 1	Survey 2	Survey 3	Survey 4
Vulnerable group				
Aboriginal and Torres Strait Islander peoples	67.2 (54; 78.7)	80.3 (68.2; 89.4)	78.4 (68.4; 86.5)	80.2 (71.5; 87.1)
People with disabilities	35.3 (30.7; 40.2)	29.7 (25.3; 34.3)	57.9 (53.6; 62.1)	54.7 (50.4; 59)
Young (<25 yrs)	42.6 (36.4; 48.9)	44.5 (38.2; 51)	47.8 (43.9; 51.8)	56.5 (52.5; 60.5)
CaLD	35.5 (31.3; 39.9)	28.9 (24.7; 33.4)	41.7 (37.7; 45.7)	47.7 (43.4; 51.9)
Low income (< $40,000 per year)	32.4 (28.3; 36.7)	29.5 (25.5; 33.8)	54.8 (50.8; 58.8)	57.7 (53.6; 61.7)
Unemployed	45.7 (37.2; 54.3)	34.9 (27.3; 43)	57.7 (50.4; 64.7)	68.3 (60; 75.7)
People with any vulnerability above	32.5 (29.9; 35.3)	29.6 (27; 32.3)	45.6 (43.2; 48)	49.9 (47.5; 52.4)
People without any vulnerability above	15.5 (13; 18.2)	13.9 (11.6; 16.4)	24.2 (21.3; 27.3)	30 (27; 33.2)

## 4 Discussion

The results of our study showed how different subgroups showed different patterns of change over time. The results that people with disabilities had poorer outcomes and were more impacted by the pandemic are similar to other research examining mental health outcomes for this cohort during the pandemic (28). Previous research has noted significant variability in one-off surveys of mental health during the pandemic and suggested that subgroups would have considerable differences but did not follow these groups over time (1). Some studies found that relative to measures taken before the pandemic, certain groups had a larger overall decline. Botha et al. (2) found that employed people had a steeper decline in mental health relative to the unemployed. However, the unemployed still had on average lower levels of mental health when surveyed during the pandemic relative to the employed sample. The comparison data prior to the pandemic were much worse, hence the smaller decline. Their study also found men and younger Australians had a steeper decline in mental health status. The results from our study suggested that, as a group, people who were unemployed had a similar pattern of change in each survey to other groups. That is, the pattern of change—either increasing or decreasing—was similar, but at each survey time point, their results were much worse than people without any vulnerability.

The results of this study showed higher rates of psychological distress among young people relative to the rest of the sample, with approximately a quarter of the sample in this category at each survey period. This is still much lower than the figure recorded by Li et al. (6) who found that close to half the sample had high psychological distress. The difference here could be twofold: one is that Li et al.’s study was conducted with adolescents and that their study was a convenience sample. An interesting parallel between our study and the research of Li et al. (6) is that, in their study, there was no difference between the results of the Victorian sample and the rest of the country, even though at the time of data collection, Victoria was the only state in lockdown. In our study, there was consistency in psychological distress for young people in each survey, irrespective of whether a lockdown was occurring or not, even in survey 2 during the longest lockdown. Thus, unlike other social connection and wellbeing variables, levels of psychological distress did not fluctuate in any consistent pattern alongside changes to government policies. It had been postulated that government restrictions may lead to boredom and reduced social contact, which will result in increased psychological distress (10). These results showed that social connection and general life satisfaction did correlate with levels of restrictions, but psychological distress did not.

The findings echo other population research conducted during the pandemic, suggesting that changes to social and work functioning themselves are associated with mental health, rather than exposure to the virus (4). The results of this study were similar to other research where social connection and wellbeing have been impacted by COVID-19-related policies (4, 7, 8). What this study adds is how social connection and wellbeing rebounded in 2022 as government restrictions were eased. It also showed how financial hardship and psychological distress increased for some, potentially due to changes to the JobKeeper payment, which again supports other research in highlighting the risk that those more vulnerable are at increased risk of financial stress and worse mental health outcomes as a result of changes to government policy (29). It corroborates other research showing how changes to the JobKeeper allowance were correlated with changes in financial stress and mental distress, suggesting the protective role it played early in the pandemic (2).

The results highlighted some interesting data on Aboriginal and Torres Strait Islander peoples that corroborate with other studies on this community during COVID-19. There was a reduction in the number of people experiencing low to medium life satisfaction from 2020 to 2022, but an increase in the number of people experiencing high psychological distress. The overall wellbeing was fairly stable, with social connection remaining high relative to other groups. However, financial hardship jumped from survey 1 and remained high in each survey period. Some of these patterns indicate there are probably within-group differences within Aboriginal and Torres Strait Islander peoples that could explain some of these inconsistencies in changes over time. Understanding these within-group differences is important from a policy perspective, with previous research highlighting how age, financial instability, and mental health comorbidity influenced self-reported health outcomes prepandemic and during the pandemic (16). One of the limitations of this study was the small sample size of Aboriginal and Torres Strait Islander peoples, which did not permit an intersectionality analysis of different demographic groupings within this community. Further research is required on how changes over time in mental health within the Aboriginal and Torres Strait Islander communities differ according to other key characteristics related to age, gender, employment status, disability, and other key demographic factors.

Another limitation of this research was that it did not explore the positive aspects of the pandemic from a qualitative perspective. Previous research has found that people nominated more time with family, workplace flexibility, and a calmer life as benefits that emerged during COVID-19 (12, 30). These are complex dynamics where there is some potential to experience positive benefits while still feeling that the overall experience is negative (12). Qualitative research may have uncovered some of the reasons why certain variables changed while others did not. Additionally, the study was limited to a cross-sectional survey with small sample sizes for some of the subgroups. This meant that longitudinal analysis based on repeat data was not possible. Further research could include larger sample sizes to enable longitudinal data to explore some of the possible relationships these data reveal. A further limitation was that no data collection was undertaken in 2021, which would have been interesting to see if the trends were smooth, as the change recorded from 2020 to 2022 was somewhat abrupt for some of the measures in this study. Further data collection in 2021 would have been particularly interesting in the Victorian context, given the long lockdowns experienced, which other states in Australia did not experience to the same degree in 2021. Data from 2021 would have further aided the analysis to determine whether changes were gradual over time as people adapted to the pandemic or if there was an abrupt change based on policy changes. The strength of the study, however, was that it was a representative sample weighted to population norms, with some of the previous variations in mental health status that resulted from convenience samples being used.

## 5 Conclusion

This study has provided a unique perspective on how different population groups fared during the pandemic with respect to mental health and social wellbeing. While there had been previous research on these groups, it often compared survey results taken before the pandemic to one survey result during the pandemic. Furthermore, many of the surveys were limited to convenience samples, which explains some of the large variation in findings. The results from this study highlight how different variables showed different patterns of results for different communities. The lockdowns seemed to impact social connections and overall life satisfaction, with better results in 2022 than in 2020 for most groups. Psychological distress and financial hardship showed a different pattern of results over time, with worse results in 2022 than in 2020, and it is interesting to speculate what impact the removal of the JobKeeper payment had on these findings. However, the fact that all groups showed an increase in financial hardship would suggest that broader economic factors were important contributors to these findings. The results also highlight the large discrepancy in mental health and social wellbeing between those with and without vulnerability. The differences between groups were often much larger than any change in results between the survey periods. Thus, irrespective of the pandemic, this highlights the ongoing need for policies that can address these large inequities in health outcomes.

## Data availability statement

The datasets presented in this article are not readily available because the data is owned by VicHealth. Requests to access the datasets should be directed to https://www.vichealth.vic.gov.au/.

## Ethics statement

Ethics approval for Surveys One and Two was provided by the Australian National University Human Research Ethics Committee (2020/264) on 20 May 2020. Ethics approval for Surveys Three and Four was provided by the Bellberry Human Research Ethics Committee (2021-11-1312) on 6 January 2022. The studies were conducted in accordance with the local legislation and institutional requirements. The participants provided their written informed consent to participate in this study.

## Author contributions

AJ: Conceptualization, Formal analysis, Writing – original draft, Writing – review & editing. TT: Conceptualization, Formal analysis, Writing – review & editing. RS: Writing – original draft, Writing – review & editing. JF: Writing – review & editing.
